# Giant lipoma pediculated in the falciform ligament: A case report

**DOI:** 10.22088/cjim.12.0.471

**Published:** 2021

**Authors:** Mostafa Sadeghi, Sajad Noorshafiee, Shaghayegh Beshtar, Paiman Rezagholy, Alireza Tavassoli

**Affiliations:** 1 Montaserieh Dialysis and Transplant Center, Mashhad University of Medical Sciences, Mashhad, Iran; 2 Endoscopic and Minimally Invasive Surgery Research Center, Mashhad University of Medical Sciences, Mashhad, Iran; 3 School of Paramedical Sciences, Iran University of Medical Sciences, Tehran, Iran; 4 Faculty of Nursing and Midwifery, Kurdistan University of Medical Sciences, Sanandaj, Iran

**Keywords:** Ligament Falciform, Lipoma, Abdominal mass

## Abstract

**Background:**

The lipoma is one of the benign soft tissue tumors that occur most in adulthood. These tumors are one of the common tumors of the limbs, and rarely occur in the abdominal cavity. These masses usually grow slowly and are asymptomatic.

**Case Presentation:**

In this article, we present a 23-year-old woman who was referred to a doctor with complaints of abdominal pain and enlargement that occurred in the last 4 months. Following surgery and sampling, it was found that the patient had a lipoma, and the liver was a phlegmatic liver in the liver form.

**Conclusion:**

The recurrence and metastasis of the primary tumor histology are not always predictable, and the authors stated that all cases of individual fibrous tumors should be known as potentially malignant.

Lipoma is one of the benign soft tissue tumors that occur most in adulthood. These tumors are one of the common tumors of the limbs, and rarely occur in the abdominal cavity. Abdominal lipomas with the origin of feline ligand liver form have rarely been reported. According to studies done so far, only three cases of lipoma of the origin of flasia ligand have been reported (1). These tumors are usually single, moving, painless, and slowly growing. If they are small, they are asymptomatic and usually have good prediction.Typically, these tumors do not require special treatment. If they are symptomatic, they should be completely removed from the body through surgery (4, 5). In this study, we report a case of abdominal lipoma, the pedicle of which is in the form of falsy ligaments. 

## Case presentation

A 23-year-old woman with cesarean section presented with big belly swelling in the past four months and initially thought to have become obese but gradually had a vague abdominal discomfort in the abdomen and therefore referred to a doctor. In a large examination of the abdominal cavity from the pelvis to the underlying gossifoid, it was similar to the term pregnancy. For more examinations, the patient was under CT angiography. 

**Figure 1:**
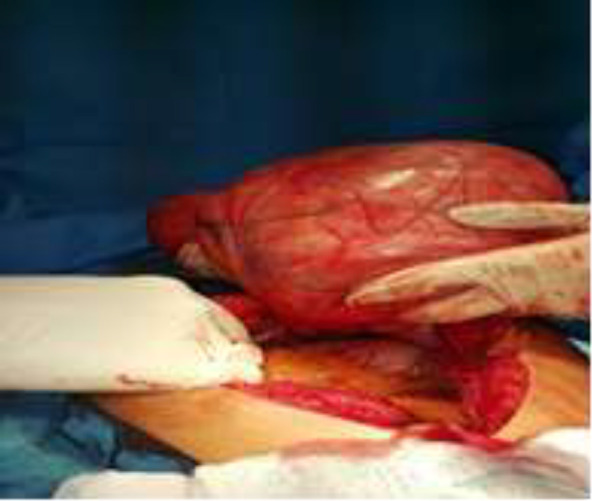
Image during surgery when mass is removed

**CT Angiography: **The intranasal peritoneal mass with a fat density and a delicate interior sponge with dimensions of 230 to 280 mm without clear anagram vascular bed and a compressive effect on the abdominal vascular vessels was observed. Hypervices in contrast postures in myometric tissue is probably related to the tissue of the tuber either (adenomyosis) or degenerative myoma. Further examination with ultrasound is recommended. Operational findings:

The abdomen was opened by midline incision and the viscera was shifted to the peritoneum in the lower right side, and the bulk mass with anterior fat and circular was measured with a size of 28 * 23 * 28 cm, which did not permeate the adhesion and was determined after release. The pedicle was thrown in the ligament, and along with the ligament throw, the phallic ligament of the form was completely removed and sent to pathology department (figure 1). The final pathology report of a large lobule mass with a total dimensions of 14 * 23 * 28 cm with a surface of perforated and flattened surface, and a plain capsule in an area in the range of 10 * 25 centimeters and in the midnight examination, sections of the proliferation of adult adipose tissue are associated with the connective tissue of areas of fat necrosis.

## Discussion

In liver ligands, rarely lesions such as lipoma, paraganglioma, lymphangioma, liposarcoma, lymosarcoma, endodermal sinus tumors can occur (1 and 2). Lipoma is one of the benign soft tissue tumors that may appear in mediastinum, retroperitoneum and pelvis, but is often seen in the limbs. Depending on where you are located, you may experience various complications such as abdominal pains, obstruction, bloating, and constipation (9, 10). These tumors are rarely created in the abdominal cavity and are usually single, moving, painless and growing at a slow pace. Due to the low growth rates and clinical manifestations of these tumors, they are often diagnosed during examinations when the patient visits a doctor for another reason (4, 5).

The duration of growth in the present patient was only 4 months long. The enlarged abdominal mass from the pelvis to the underlying gossifoid was symmetrical and looked like term pregnancy and its origin was in in the liver in the liver form. Due to various proteins that other soft tissue tumors, such as liposarcoma, angioiloma, and trematoma, differential diagnosis of intra-abdominal lesions is of particular importance. In radiologic images, the lipoma is observed in hypercogenetic masses with low edges and posterior bases and may be mistaken for hemangiomas, except that the hemangioma has a posterior thoracic base. Sometimes the nature and origin of such lesions cannot be detected by CT scan. For this purpose, we must use laparotomy and pathological examination of the sample (2).

In the present case, imaging techniques were not able to differentiate the lipoma from adenomyosis or degenerative myoma. Liver ligament tumors of the liver form are rare. Leiomyosarcomas, lipoma and endodermal sinus have been reported to appear in the form of Falcius ligament (11).

Solitary fibrous tumors of the bone in several places, including the abdomen have been reported. In a study, 10 of 92 abnormal soluble fibroblastomas that were clinically or pathologically malignant were identified. The recurrence and metastasis of the primary tumor histology are not always predictable, and the authors stated that all cases of individual fibrous tumors should be known as potentially malignant (12).

## References

[1] Papachristou E, Provatopoulou S, Savvidaki E (2014). Outcome of Transplantation in Renal Allograft Recipients from cadaveric donors with standard and expanded criteria: a single-center experience. Transplantat Proc.

[2] Lagoudianakis EE, Michalopoulos N, Markogiannakis H (2008). A symptomatic cyst of the ligamentum teres of the liver: A case report. World J Gastroenterol.

[3] Gidwani AL, Mullan FJ, Kenny B (2004). Solitary fibrous tumour of the falciform ligament containing multiple foci of malignant transformation. J Clin Pathol.

[4] Joji Reddy O, Abdul Gafoor J, Suresh B, Obuleswar Prasad P (2015). Lipoma in liver: A rare presentation. J NTR Univ Health Sci.

[5] Özer M, Ulusoy S, Parlak Ö (2016). A Rare location: a giant mesenteric lipoma. Med J Islamic World Acad Sci.

[6] Kakitsubata Y, Nakamura R, Shiba T (1993). Lipoma of the falciform ligament: US, CT, and MRI appearances. Clin Imaging.

[7] Honda H, Watanabe K, Mihara K, Hoshi H, Sakihama M (1983). Lipoma of the hepatic falciform ligament. J Comput Assist Tomogr.

[8] Lagoudianakis EE, Michalopoulos N, Markogiannakis H (2008). A symptomatic cyst of the ligamentum teres of the liver: A case report. World J Gastroenterol.

[9] Ozel SK, Apak S, Ozercan IH, Kazez A (2004). Giant mesenteric lipoma as a rare cause of ileus in a child: report of. Surg Today.

[10] Cherian A, Singh SJ, Broderick N, Zaitoun AM, Kapila L (2004). Small bowel volvulus due to giant mesenteric lipoma. Pediatr Surg Int.

[11] Morgan K, Ricketts RR (2004). Lymphangioma of the falciform ligament—a case report. J Pediatr Surg.

[12] Gold JS, Antonescu CR, Hajdu C (2002). Clinicopathologic correlates of solitary fibrous tumors. Cancer.

